# Associations Between *DAT1* Gene VNTR Polymorphism and Impulsivity Dimensions in Individuals with Behavioural Addictions

**DOI:** 10.3390/biomedicines13081852

**Published:** 2025-07-30

**Authors:** Remigiusz Recław, Aleksandra Suchanecka, Elżbieta Grzywacz, Krzysztof Chmielowiec, Jolanta Chmielowiec, Anna Makarewicz, Kinga Łosińska, Dariusz Larysz, Grzegorz Trybek, Anna Grzywacz

**Affiliations:** 1 Independent Laboratory of Behavioral Genetics and Epigenetics, Pomeranian Medical University in Szczecin, Powstancow Wielkopolskich 72 St., 70-111 Szczecin, Poland or remigiusz.reclaw@awf.gda.pl (R.R.); aleksandra.suchanecka@pum.edu.pl (A.S.); 2 Department of Medical Sciences and Public Health, Gdansk University of Physical Education and Sport, Kazimierza Gorskiego 1 St., 80-336 Gdansk, Poland; kinga.losinska@awf.gda.pl (K.Ł.); grzegorz.trybek@pum.edu.pl (G.T.); 3 Doctoral School, Pomeranian Medical University in Szczecin, Powstancow Wielkopolskich 72 St., 70-111 Szczecin, Poland; elagrzywacz@me.com; 4 Department of Hygiene and Epidemiology, Collegium Medicum, University of Zielona Góra, 28 Zyty St., 65-046 Zielona Góra, Poland; chmiele@vp.pl (K.C.); chmiele1@o2.pl (J.C.); makarewicz81@gmail.com (A.M.); 5 109th Military Hospital with Polyclinic, Ministry of National Defense, ul. Ksiedza Piotra Skargi 9/11, 71-422 Szczecin, Poland; dariuszlarysz@hotmail.com; 6 Department of Interdisciplinary Dentistry, Pomeranian Medical University in Szczecin, Powstancow Wielkopolskich 72 St., 70-111 Szczecin, Poland; 7 Maxillofacial Surgery Clinic, 4th Military Clinical Hospital in Wroclaw, Rudolfa Weigla 5 St., 50-981 Wroclaw, Poland

**Keywords:** *DAT1*, impulsivity, behavioural addictions, VNTR polymorphism, dopamine transporter

## Abstract

**Background/Objectives**: Impulsivity is a key psychological construct implicated in the onset and maintenance of behavioural addictions. Dysregulation of impulsivity is central to behavioural addictions, yet its genetic basis remains unclear. This study examined the association between the *DAT1* variable number tandem repeat polymorphism and impulsivity in individuals with behavioural addictions. **Methods**: A total of 328 males (128 with behavioural addictions and 200 controls) completed the Barratt Impulsiveness Scale. *DAT1* genotyping was performed via PCR and gel electrophoresis. Statistical analyses included chi-square tests, Mann–Whitney U-tests, and two-way ANOVA. **Results**: No differences in *DAT1* genotype frequencies were found between groups. However, a significant interaction emerged for attentional impulsivity: individuals with behavioural addictions and the 9/9 genotype had the highest BIS-AI scores (F_2, 322_ = 5.48; *p* = 0.0046). **Conclusions**: The *DAT1* 9/9 genotype may increase vulnerability to attentional impulsivity, but only in the context of behavioural addictions. These findings highlight a gene–environment interaction and support the role of dopaminergic mechanisms in cognitive dysregulation. Future studies should validate these findings using longitudinal designs and neurobiological methods.

## 1. Introduction

Impulsivity is a multidimensional psychological construct encompassing tendencies toward rapid, unplanned reactions to internal or external stimuli without regard to the negative consequences [1]. It plays a pivotal role in numerous psychopathological conditions, including attention-deficit/hyperactivity disorder (ADHD), substance use disorders, and behavioural addictions [2]. From a neurobiological perspective, impulsivity has been closely linked to the dopaminergic system, particularly the function of dopamine transporters, which regulate synaptic dopamine availability and thereby modulate behavioural control and reward sensitivity [3].

The dopamine transporter gene (*DAT1* or *SLC6A3*), located on chromosome 5p15.3, contains a 40-base pair variable number tandem repeat (VNTR) polymorphism in its 3′-untranslated region (3′UTR) [4]. The two most common allelic variants are the 9-repeat (9R) and 10-repeat (10R) alleles [5], which have been found to influence the expression and availability of dopamine transporters. Although evidence remains mixed, some studies have suggested that the 10R allele is associated with higher *DAT* expression and more efficient dopamine reuptake, which may relate to traits such as reduced cognitive flexibility or increased vulnerability to impulsive decision-making [6]. Conversely, the 9R allele has been associated with altered reward processing and heightened sensitivity to reinforcement cues [7].

Behavioural addictions, including compulsive gaming, pornography use, and social media overuse, are increasingly recognised as clinically significant disorders [8]. These behaviours share core characteristics with substance addictions, including impaired control, compulsivity, craving, and continued engagement despite adverse outcomes [9]. Importantly, they also exhibit strong associations with heightened impulsivity, particularly domains of attention and motor control. However, unlike substance use disorders, the genetic basis of behavioural addictions remains underexplored, and studies examining specific candidate genes, such as *DAT1*, are scarce.

Most previous genetic studies have focused on impulsivity in the context of externalising disorders or substance dependence [10,11]. Very few have addressed the interaction between dopamine-related genetic variants and behavioural addictions, especially concerning specific impulsivity subdomains measured by validated psychometric instruments [12]. Moreover, much of the existing literature fails to consider the interaction between genetic susceptibility and addiction status, often analysing genotypic effects across heterogeneous or non-clinical populations [12].

The Barratt Impulsiveness Scale (BIS-11) was selected for this study due to its robust psychometric properties and its long-standing use in both clinical and neurogenetic research [13,14]. The BIS-11 enables the assessment of three theoretically grounded subcomponents of impulsivity—attentional, motor, and non-planning—allowing for a more nuanced understanding of how genetic variation may differentially affect cognitive and behavioural dimensions of impulse control [14]. Its sensitivity to subtle intergroup and genotype-related differences makes it particularly well-suited for studies exploring gene–behaviour interactions [13].

This study aims to address this gap by investigating whether *DAT1* VNTR polymorphisms are associated with impulsivity in individuals diagnosed with behavioural addictions, and whether these associations differ from those observed in healthy controls [11]. By focusing on the Barratt Impulsiveness Scale (BIS-11) and analysing genotype × group interactions, we seek to elucidate the modulatory role of dopamine transporter variation in cognitive aspects of impulsivity [15]. Understanding these relationships may not only improve our conceptualisation of behavioural addiction vulnerability but also inform more individualised approaches to prevention and treatment [11,15].

## 2. Materials and Methods

### 2.1. Study Participants

The study included 328 male participants: 128 with a diagnosis of behavioural addiction and 200 individuals assigned to the control group. All participants provided written informed consent. Recruitment and diagnostic procedures were conducted in a therapeutic facility for individuals with behavioural addictions (study group) and among candidates volunteering for the control group, selected from the general population of young adult males aged 18–30.

Psychiatric evaluation and group assignment (behavioural addiction vs. control) were performed using the structured Mini International Neuropsychiatric Interview (MINI). Additionally, the Barratt Impulsiveness Scale (BIS-11) and the Adult ADHD Self-Report Scale (ASRS v1.1) were administered.

The inclusion criteria for the behavioural addiction group were as follows: male sex, age over 18 years, and a clinical diagnosis of behavioural addiction confirmed through an interview. The control group consisted exclusively of males without current or past psychiatric diagnoses, including any form of addiction.

Exclusion criteria for both groups included intellectual disability, dementia, psychoactive substance use disorder, neurodevelopmental disorders, history of traumatic brain injury, current suicide risk, and clinically significant somatic conditions (e.g., cardiovascular, endocrine, neurological, or metabolic disorders) that could affect cognitive functioning or mental health.

The study protocol was approved by the local Bioethics Committee (KB-0012/106/16).

### 2.2. Measures

**Barratt Impulsiveness Scale (BIS-11):** Impulsivity was measured using the Barratt Impulsiveness Scale, version 11 (BIS-11), a widely used self-report questionnaire designed to assess the personality and behavioural construct of impulsiveness [13]. The BIS-11 consists of 30 items rated on a 4-point Likert scale ranging from 1 (“Rarely/Never”) to 4 (“Almost Always/Always”) [13]. The questionnaire yields a total impulsivity score and three primary subscale scores: Attentional Impulsivity (AI), which reflects difficulties in focusing and cognitive instability; Motor Impulsivity (MI), which pertains to acting without thinking; and Non-Planning Impulsivity (NPI), which relates to a lack of forethought and future orientation [13]. The BIS-11 has demonstrated good psychometric properties, including internal consistency and construct validity, across various populations and cultural contexts [13]. Higher scores indicate greater levels of impulsivity. In this study, the BIS-11 was administered in its Polish adaptation, which has been validated and standardised for use in clinical and non-clinical Polish-speaking populations.

The Adult ADHD Self-Report Scale (ASRS v1.1) is a symptom checklist that consists of the eighteen DSM-IV-TR criteria. Six of the eighteen questions were found to be the most predictive of symptoms consistent with ADHD [16].

### 2.3. Genotyping

Genomic DNA was extracted from peripheral venous blood samples using standard phenol–chloroform procedures, following established laboratory protocols to ensure high purity and yield (Roche Diagnostics, Mannheim, Germany). The concentration and quality of the isolated DNA were assessed spectrophotometrically.

The *DAT1* genotypes were categorised based on the presence of 9- and 10-repeat alleles. Genotyping was conducted using the PCR technique with the following primers: forward 5′-TGT GGT GTA GGG AAC GGC CTG AG-3′ and reverse 5′-CTT CCT GGA GGT CAC GGC TCA AGG-3′. Each 25 μL reaction mixture contained 100 ng of genomic DNA, 10 pmol of each primer, 50 mM KCl, 10 mM Tris-HCl, 1.5 mM MgCl_2_, 200 μM each dNTP (dATP, dCTP, dTTP, dGTP), and 0.8 U of Taq DNA polymerase. The PCR programme included an initial denaturation at 94 °C for 5 min, followed by 30 cycles of denaturation at 94 °C for 55 s, primer annealing at 55 °C for 50 s, and extension at 72 °C for 1 min, ending with a final elongation step at 72 °C for 10 min. Amplified DNA fragments were separated by electrophoresis on a 3% agarose gel stained with ethidium bromide and visualised under UV light. The expected product sizes were 450 bp for the 10-repeat allele and 410 bp for the 9-repeat allele (Supplementary Figure S1) [17].

### 2.4. Statistical Analysis

A concordance between the genotype frequency distribution and Hardy–Weinberg equilibrium (HWE) was tested using the online HWE calculator (https://wpcalc.com/en/equilibrium-Hardy-Weinberg/, accessed on 19 June 2025).

Genotype frequencies between individuals with behavioural addictions and healthy control subjects were compared using the chi-square test.

The relationship between *DAT1* gene variants and impulsivity was assessed using multivariate analysis of variance (ANOVA). The factorial ANOVA model included two main factors: BIS-11 subscale (attentional, motor, and non-planning), group status (behavioural addictions vs. control), and their interaction (subscale × genotype × group). Before conducting parametric tests, the assumption of homogeneity of variance was verified using Levene’s test, which confirmed equality of variances (*p* > 0.05). As the distribution of analysed variables did not meet the criteria for normality, nonparametric comparisons were also performed using the Mann–Whitney U test for independent samples.

All statistical analyses were conducted using STATISTICA 13 (TIBCO Software Inc., Palo Alto, CA, USA) running on Microsoft Windows (Microsoft Corporation, Redmond, WA, USA). Effect sizes for ANOVA results were reported using eta-squared (η^2^).

## 3. Results

In our sample, the majority of study participants were single (89.1% in the case group and 87% in the control group), lived in the village (75.8% in the case group and 69.5% in the control group), and had a high school-level education (39.8% in the case group and 56% in the control group). However, the case group was significantly younger (mean = 27.73 years in the case group vs. mean = 22.18 years in the control group; Z = 10.249; *p*< 0.0001), which should be taken into consideration (Table 1).

Genotype distributions for the *DAT1* VNTR polymorphism in both the behavioural addictions group and the control group conformed to Hardy–Weinberg equilibrium (Table 2).

The distribution of *DAT1* genotypes—9/9, 9/10, and 10/10—did not differ significantly between individuals with behavioural addictions and controls (χ^2^ = 3.349, *p* = 0.1874). Similarly, allele frequencies (9R vs. 10R) did not show significant differences between groups (χ^2^ = 0.0127, *p* = 0.9101) (Table 3).

Descriptive statistics for psychological measures, including the BIS-11 total score and its subscales (Attentional Impulsivity [AI], Motor Impulsivity [MI], and Non-Planning Impulsivity [NI]), are presented in Table 4.

Participants with behavioural addictions scored significantly higher than controls on the BIS total score (Z = 4.203, *p* < 0.0001), as well as on the Attentional Impulsivity (AI) subscale (Z = 4.455, *p* < 0.0001) and the Motor Impulsivity (MI) subscale (Z = 4.809, *p* < 0.0001) (Table 4).

A two-way ANOVA was conducted to evaluate the main and interaction effects of group status (addiction vs. control) and *DAT1* genotype on psychological measures. The results are summarised in Table 5.

A significant main effect of group status was found for Attentional Impulsivity (F_1, 322_ = 19.09, *p* < 0.0001, η^2^ = 0.056), indicating that individuals with behavioural addictions experienced more pronounced difficulties with attention regulation compared to controls.

Crucially, a significant interaction effect between *DAT1* genotype and group status was observed for Attentional Impulsivity (F_2, 322_ = 5.48, *p* = 0.0046, η^2^ = 0.033), suggesting that the relationship between genotype and impulsivity depends on addiction status. Post hoc analyses indicated that among individuals with behavioural addictions, those with the 9/9 genotype had significantly higher AI scores compared to carriers of the 9/10 and 10/10 genotypes. These post hoc comparisons are detailed in Table 6.

**Figure 1:**
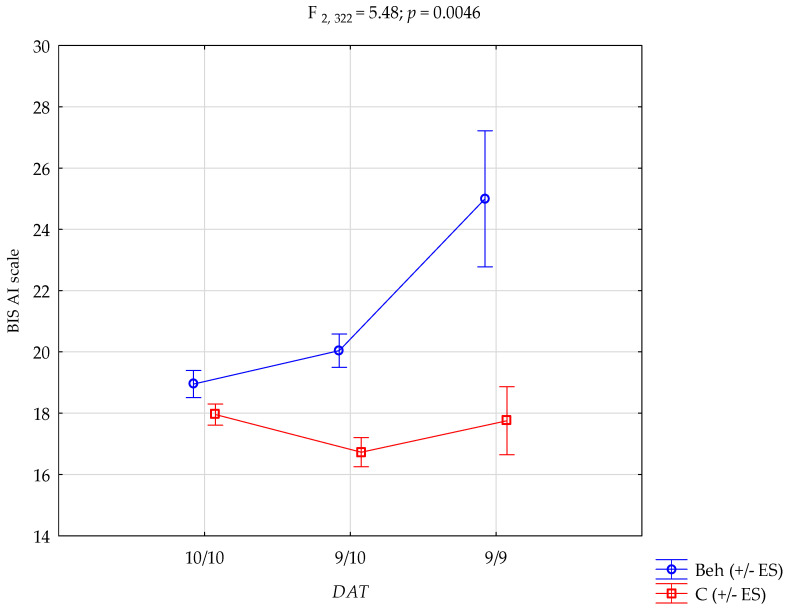
*DAT1* × group interaction in attentional impulsivity. Beh—behavioural addictions, C—Control; +/− ES—standard error.

The genotype × group interaction on Attentional Impulsivity is graphically presented in Figure 1. The plot illustrates that the highest AI scores were observed in individuals with behavioural addictions in the group with the 9/9 genotype, whereas the genotype had little effect in the control group. This visualisation highlights the conditional expression of genetic vulnerability, supporting a gene-by-environment interaction model. Together, these results emphasise that while the *DAT1* VNTR polymorphism alone does not differentiate individuals with behavioural addictions from controls, its interaction with addiction status significantly modulates multiple dimensions of impulsivity, underscoring the conditional expression of genetic vulnerability in the context of behavioural addictions.

Table 6 presents the results of the post hoc analysis. Participants with behavioural addictions carrying the 10/10 genotype had significantly lower BIS-AI scores compared to controls with the 9/9 genotype. Behavioural addiction patients with the 9/10 genotype scored significantly lower on the BIS-AI scale than those with the 9/9 genotype within the same group. Moreover, behavioural addiction patients with the 9/10 genotype had significantly higher BIS-AI scores than controls with either the 10/10 or 9/10 genotypes. Individuals with behavioural addictions and the 9/9 genotype scored significantly higher on BIS-AI than all control subgroups (10/10, 9/10, and 9/9). Finally, within the control group, those with the 10/10 genotype showed significantly higher BIS-AI scores than those with the 9/10 genotype.

## 4. Discussion

The present study explored the relationship between *DAT1* VNTR polymorphisms and impulsivity dimensions in individuals with behavioural addictions, providing evidence of a significant gene-by-group interaction effect on attentional impulsivity (AI). While no differences were found in *DAT1* genotype or allele frequencies between addicted and control groups, individuals with behavioural addictions who were homozygous for the 9R allele (9/9) exhibited the highest scores on the AI subscale of the BIS-11. These findings highlight the conditional influence of *DAT1* variation on cognitive control mechanisms and suggest that the 9/9 genotype may confer greater susceptibility to attentional dysregulation in the context of behavioural addictions.

Our results align with previous research [18,19,20] linking the 9R allele to increased sensitivity to reward and dysregulation in dopaminergic pathways, particularly those involving the mesolimbic and mesocortical systems. Prior studies [21] have demonstrated associations between the 9R allele and higher levels of impulsive decision-making and novelty seeking in both clinical and non-clinical populations. The current findings expand upon this literature by demonstrating that such effects may be particularly pronounced in individuals with behavioural addictions, supporting a model in which genetic predispositions interact with behavioural contexts to influence impulsivity-related traits.

Interestingly, significant interaction was observed only for attentional impulsivity, and not for the motor or non-planning subscales. This may reflect a more direct link between *DAT1* expression and prefrontal–striatal circuits implicated in sustained attention, vigilance, and executive functioning. The dopamine transporter, encoded by *DAT1*, is expressed at high levels in the striatum and regulates dopamine reuptake, thus controlling dopaminergic tone in pathways that support attentional filtering and goal-directed behaviour. The 9/9 genotype may be associated with reduced *DAT1* transcription efficiency, leading to altered dopaminergic signalling and decreased cognitive stability. As a result, individuals may experience greater distractibility and difficulty sustaining attention, particularly in environments rich in rewarding stimuli, a hallmark of many behavioural addiction settings. Importantly, *DAT1* regulation may not be purely genetic. Recent findings suggest that the methylation patterns of the *DAT1* promoter differ between stimulant-dependent individuals and controls, with specific CpG island methylation levels correlating with personality traits such as neuroticism and conscientiousness [22]. DNA methylation is one of the most studied epigenetic mechanisms. It involves the addition of a methyl group (CH_3_) to the fifth carbon of cytosine, typically within CpG dinucleotides, i.e., regions of DNA where a guanine nucleotide follows a cytosine nucleotide. These CpG sites are often clustered in genomic regions known as CpG islands, which are typically located in gene promoter regions and play a crucial role in regulating gene transcription [23]. When CpG islands become hypermethylated, gene expression is typically reduced or silenced, whereas hypomethylation is often associated with increased gene expression [24,25]. This process supports the view that impulsivity and addiction vulnerability are shaped by both genetic and epigenetic influences, a perspective with potential clinical implications for individualised prevention and treatment strategies [11].

The selective nature of the interaction effect suggests domain-specific vulnerability rather than a generalised impulsivity phenotype. While motor impulsivity is linked to behavioural disinhibition and rapid responding, attentional impulsivity reflects cognitive lapses, susceptibility to distraction, and challenges in maintaining task focus. Given that many behavioural addictions—such as internet gaming or social media overuse—involve prolonged attentional engagement with variable reinforcement, it is plausible that cognitive rather than behavioural control mechanisms are more strongly implicated in vulnerability and maintenance [26].

No significant main effect of the *DAT1* genotype was observed in the overall sample, reinforcing the notion that genetic variants do not act in isolation but exert their influence in specific environmental or behavioural contexts. This is consistent with the diathesis–stress framework, which posits that specific genotypes only become functionally relevant under particular conditions of stress or pathology [8,11]. In this case, the elevated AI scores in 9/9 carriers emerged only in the presence of behavioural addictions, suggesting a latent genetic vulnerability that is unmasked by addictive engagement.

The BIS-11, used in this study to assess impulsivity dimensions, has proven especially valuable in delineating domain-specific associations [27]. Its construct validity and cross-cultural application have made it one of the most widely adopted tools in both clinical psychology and behavioural genetics. The ability to detect fine-grained differences across impulsivity domains enhances its utility in genotype–phenotype association studies, as seen here. Interestingly, while individuals with behavioural addictions scored significantly higher on total BIS-11 scores and attentional and motor impulsivity subscales, no significant difference was observed in non-planning impulsivity. This suggests that behavioural addictions may not uniformly affect all dimensions of impulsivity. Non-planning impulsivity, which reflects a lack of future orientation and poor organisational skills, may not be a core feature of behavioural addictions in the studied population. This pattern aligns with recent findings suggesting that behavioural addictions are more closely linked to deficits in inhibitory control, behavioural inhibition, and weakened conflict control, rather than impaired long-term planning [28,29]. These findings underscore the importance of considering impulsivity as a multidimensional construct in both research and clinical practice.

These findings carry both theoretical and clinical implications. On a theoretical level, they support the view that behavioural addictions share neurobiological substrates with substance-related disorders and are modulated by common genetic pathways [29,30]. The *DAT1* VNTR polymorphism may serve as one such shared biomarker, highlighting dopaminergic mechanisms that underlie impulsivity across diagnostic boundaries. Clinically, the results underscore the importance of individualised assessments that integrate both genetic and psychological dimensions. Knowledge of genetic vulnerability may inform more tailored approaches to prevention and treatment. For example, interventions aimed at enhancing attentional control or mitigating cognitive distraction may be especially beneficial for individuals with the 9/9 genotype.

In summary, this study provides novel evidence that the *DAT1* VNTR 9/9 genotype is associated with elevated attentional impulsivity, but only in the presence of behavioural addictions. These results highlight the need to consider both genetic vulnerability and behavioural context in understanding impulsive traits and their contribution to addictive disorders. Future research should aim to replicate and expand upon these findings using longitudinal, multi-method, and integrative designs that bridge the gap between genes, brain, and behaviour.

Despite its strengths, this study has several limitations that should be acknowledged and addressed in future research. First, the sample consisted exclusively of male participants, which limits the generalizability of the findings across sexes [31].The main reason for analysing only male samples was that behavioural addictions have been consistently shown to disproportionately affect males in both clinical and population-based studies. According to Stevens et al. [32], the prevalence of gaming disorder is significantly higher in males, with a male-to-female ratio of 2.5:1 to 4:1. A meta-analysis by Gao et al. [33] found that the pooled prevalence of internet gaming disorder among adolescents and young adults was 15.4% in males vs. 6.4% in females. Additionally, sex-specific effects have been observed in dopamine-related genes and their behavioural correlates, making it essential for future research to include female participants to examine the potential moderating roles of biological sex, hormonal influences, or X-linked genetic interactions.

Second, although the sample size was adequate for detecting interaction effects, replication in larger and more demographically diverse cohorts is needed to confirm robustness and external validity. Additionally, the subgroup of individuals with the 9/9 *DAT1* genotype within the behavioural addictions group was relatively small. This may have reduced statistical power for post hoc comparisons and increased the risk of Type II errors. Replication in larger, independent cohorts is necessary to confirm the robustness and external validity of these results.

Third, this study focused solely on the 40 bp VNTR polymorphism in the 3′ untranslated region (3′UTR) of the *DAT1* gene [34]. Although this variant is among the most studied and functionally relevant, it represents only one component of the gene’s regulatory architecture. Other functional single-nucleotide polymorphisms (SNPs), intronic variations, or haplotypes may also affect *DAT1* expression and function. Additionally, epigenetic regulation was not assessed, despite its known role in modulating *DAT1* transcription and potential interaction with genetic variation to influence impulsivity traits [35].

While impulsivity was measured using a well-validated self-report scale, future studies may benefit from integrating behavioural paradigms (e.g., go/no-go tasks, delay discounting) or neuroimaging techniques (e.g., fMRI during cognitive control tasks) to more precisely characterise the neural correlates of DAT1-related impulsivity [28,36]. Longitudinal designs could also help to determine whether the *DAT1* genotype influences not only impulsivity traits but also the trajectory and severity of behavioural addictions over time. The study relied on a single, self-report instrument—the BIS-11—to assess impulsivity, which introduces subjective bias. Although BIS-11 is widely validated and commonly used in clinical and genetic research, it may not fully capture behavioural manifestations of impulsivity or their neural correlates. Future studies should integrate performance-based tasks (e.g., stop-signal reaction time, delay discounting) and neuroimaging techniques (e.g., fMRI, PET) to provide convergent and biologically grounded evidence of impulsivity traits [36].

The cross-sectional design precludes causal inference. It remains unclear whether the *DAT1* 9/9 genotype confers vulnerability to behavioural addictions through increased attentional impulsivity or whether addictive behaviours themselves shape cognitive functioning in a genotype-dependent manner. Longitudinal research is required to clarify the directionality and temporal stability of these effects.

Although all participants in the addiction group met diagnostic criteria for behavioural addictions, the group included individuals with a range of specific disorders (e.g., gaming, pornography, shopping). These subtypes may have distinct psychological and neurobiological profiles, and future studies should stratify participants accordingly to determine whether the observed gene–impulsivity associations are consistent across types of behavioural addictions [37].

The statistical approach employed standard two-way ANOVA models and did not account for potential covariates, such as age, education level, or duration of addiction. Incorporating more advanced statistical methods, including linear mixed models or structural equation modelling, could improve sensitivity and better model nested or interacting variables.

Biological or neurochemical measures (e.g., *DAT1* expression levels, dopamine concentration) were not included, limiting the ability to link genetic variation to neurobiological processes [38] directly. Without these data, interpretations of functional consequences remain inferential. Future research should explore the combined effects of genotype and gene expression, possibly through integrative multi-omic approaches.

Additionally, the findings were derived from a single national population without a replication or validation sample. This limits generalizability and increases the risk of population-specific effects or spurious findings. Independent replication in diverse samples is essential to establish the reliability of the observed associations.

A significant age difference was observed between the behavioural addictions group and the control group. This difference may have influenced the results, particularly concerning developmental differences in impulsivity and environmental exposure. Impulsivity, particularly attentional impulsivity, can vary across different developmental stages. The prefrontal cortex, which plays a key role in executive functioning and impulse control, undergoes prolonged maturation into early adulthood [39]. Younger individuals (as in the control group) may display greater behavioural disinhibition. In contrast, older individuals (as in the behavioural addictions group) may exhibit cognitive impulsivity, such as difficulty sustaining attention or filtering distractions [40,41]. Future studies should aim to match groups more closely in age or include age as a covariate in statistical models, to better isolate the effects of behavioural addiction status and genotype.

Lastly, the study did not assess cultural or environmental factors, which are critical for understanding gene–environment interactions [42]. All participants came from a relatively homogeneous cultural and linguistic context. Environmental variables such as early childhood experiences, trauma, parenting style, or socioeconomic status may substantially influence impulsivity and addiction vulnerability [43]. Including these factors in future models would allow for a more comprehensive and ecologically valid understanding of the interplay between genes and environment.

Taken together, while the current study offers important insights into the genetic modulation of attentional impulsivity in behavioural addictions, these limitations highlight the need for cautious interpretation and underscore the importance of multidimensional, interdisciplinary approaches in future research.

## 5. Conclusions

The findings of this study provide evidence that the *DAT1* VNTR polymorphism, particularly the 9/9 genotype, is associated with elevated attentional impulsivity—but only in the context of behavioural addictions. No significant differences were observed in *DAT1* genotype or allele frequencies between the behavioural addiction and control groups, suggesting that this genetic variant alone is not a direct risk factor for the development of behavioural addictions.

However, a significant interaction effect was identified between *DAT1* genotype and group status on attentional impulsivity, as measured by the Barratt Impulsiveness Scale (BIS-11). Individuals with behavioural addictions scored significantly higher on total BIS-11 scores, as well as on attentional and motor impulsivity subscales, while no differences were found in non-planning impulsivity. This pattern suggests that behavioural addictions may not uniformly affect all dimensions of impulsivity.

Individuals with behavioural addictions carrying the 9/9 genotype exhibited the highest BIS-AI scores, significantly higher than those with 9/10 or 10/10 genotypes. This effect was not present in the control group, indicating that the genetic influence on attentional impulsivity may emerge only in the presence of addictive behaviours, thus supporting a gene–environment interaction model. No significant interaction was found for motor or non-planning impulsivity, highlighting the specificity of the *DAT1* × group effect to the attentional domain of impulsivity.

The observed association between the *DAT1* 9/9 genotype and attentional impulsivity is consistent with previous research on the role of the dopamine transporter in cognitive regulation, particularly within the prefrontal–striatal circuits involved in sustained attention and executive function. The absence of this effect in controls suggests that genetic vulnerability may manifest only under specific environmental or behavioural conditions, such as those associated with behavioural addictions.

In conclusion, this study demonstrates that the *DAT1* 9/9 genotype is linked to elevated attentional impulsivity, but only among individuals diagnosed with behavioural addictions. These findings support the conditional expression of genetic predisposition and underscore the importance of integrating genetic, psychological, and behavioural perspectives in the study of behavioural addictions.

## Figures and Tables

**Table 1:** Demographic data of behavioural addictions and the control group.

		Behavioural Addicts		Control	
		*n* = 128	%	*n* = 200	%
Marital status	Single	114	89.1	174	87
	Married	7	5.5	20	10
	Divorced	6	4.7	5	2.5
	Widowed	1	0.8	1	0.5
Education	Primary	9	7.0	5	2.5
	Vocational	39	30.5	58	29.0
	High school	51	39.8	112	56.0
	Higher	29	22.7	25	12.5
Residence	City	31	24.2	61	30.5
	Village	97	75.8	139	69.5

**Table 2:** Hardy–Weinberg equilibrium in both groups.

Hardy–Weinberg Equilibrium Including Analysis for Ascertainment Bias		Observed (Expected)	Allele Freq	χ^2^ (*p* Value)
Behavioural addictions *n* = 128	10/10	75 (78.1)	*p* (10)= 0.78 q (9)= 0.22	2.612 (0.1060)
	9/10	50 (43.8)		
	9/9	3 (6.1)		
Control *n* = 200	10/10	123 (120.9)	*p* (10)= 0.78 q (9)= 0.22	0.736 (0.3910)
	9/10	65 (69.2)		
	9/9	12 (9.9)		

*p*—statistical significance (Chi-square test); *n*—number of subjects.

**Table 3:** *DAT1* genotype and allele frequencies in behavioural addictions and control groups.

*DAT*					
	Genotypes			Alleles	
	10/10 *n* (%)	9/10 *n* (%)	9/9 *n* (%)	10 *n* (%)	9 *n* (%)
Behavioural addictions *n* = 128	75 (58.59%)	50 (39.06%)	3 (2.34%)	200 (78.12%)	56 (21.88%)
Control *n* = 200	123 (61.50%)	65 (32.50%)	12 (6.00%)	311 (77.75%)	89 (22.25%)
χ^2^ (*p* value)	3.349 0.1874			0.0127 (0.9101)	

*p*—statistical significance (Chi-square test); *n*—number of subjects.

**Table 4:** Scores on the Barratt Impulsiveness Scale (BIS-11) in healthy controls and individuals with behavioural addictions.

BIS-11 Scale	Behavioural Addictions (*n* = 128) M ± SD	Control (*n* = 200) M ± SD	Z	*p*-Value
BIS-AI	19.52 ± 4.34	17.54 ± 3.59	4.455	0.0001 *
BIS-MI	26.00 ± 4.83	23.46 ± 4.28	4.809	0.0001 *
BIS-NI	27.72 ± 4.83	27.07 ± 4.21	1.367	0.1717
BIS-11 TOTAL	73.23 ± 12.00	67.98 ± 10.16	4.203	0.0001 *

*p*—statistical significance (Mann–Whitney U test); *n*—number of subjects; M ± SD—mean ± standard deviation; *—statistically significant differences.

**Table 5:** Two-way ANOVA results for BIS-11 subscales and total scores by *DAT*1 genotype and diagnostic group.

BIS-11 Scale	Group	*DAT*				ANOVA		
		10/10 *n* = 198 M ± SD	9/10 *n* = 115 M ± SD	9/9 *n* = 15 M ± SD	Factor	F (*p* Value)	η^2^	Power (Alfa = 0.05)
BIS-AI	Behavioural addictions (Beh); *n* = 128	18.95 ± 4.19	20.04 ± 4.40	25.00 ± 2.00	Intercept Beh/control *DAT* Beh/control × *DAT*	F_1, 322_ = 1934.90 (*p* < 0.0001) * F_1, 322_ = 19.09 (*p* < 0.0001) * F_2, 322_ = 2.75 (*p* = 0.0654) F_2, 322_ = 5.48 (*p* = 0.0046) *	0.857 0.056 0.017 0.033	1.000 0.992 0.541 0.848
	Control; *n* = 200	17.95 ± 3.60	16.72 ± 3.55	17.75 ± 3.22				
BIS-MI	Behavioural addictions (Beh); *n* = 128	25.71 ± 4.78	26.16 ± 4.93	30.67 ± 2.31	Intercept Beh/control *DAT* Beh/control × *DAT*	F_1, 322_ = 2411.77 (*p* < 0.0001) * F_1, 322_ = 17.48 (*p =* < 0.0001) * F_2, 322_ = 0.91 (*p* = 0.4048) F_2, 322_ = 2.01 (*p* = 0.1353)	0.882 0.051 0.006 0.012	1.000 0.986 0.206 0.414
	Control; *n* = 200	23.55 ± 4.48	23.45 ± 3.80	22.58 ± 4.87				
BIS-NI	Behavioural addictions (Beh); *n* = 128	27.28 ± 4.71	28.36 ± 5.01	28.00 ± 5.29	Intercept Beh/control *DAT* Beh/control × *DAT*	F_1, 322_ = 2880.72 (*p* < 0.0001) * F_1, 322_ = 0.64 (*p =* 0.4257) F_2, 322_ = 0.05 (*p* = 0.9541) F_2, 322_ = 1.63 (*p* = 0.1973)	0.899 0.002 0.0003 0.010	1.000 0.125 0.057 0.344
	Control; *n* = 200	27.35 ± 4.35	26.51 ± 3.55	27.33 ± 5.85				
BIS-11 TOTAL	Behavioral addictions (Beh); *n* = 128	71.93 ± 11.76	74.54 ± 12.27	83.67 ± 8.33	Intercept (Beh)/control *DAT* (Beh)/control × *DAT*	F_1, 322_ = 3347.91 (*p* < 0.0001) * F_1, 322_ = 13.10 (*p =* 0.0003) * F_2, 322_ = 1.10 (*p* = 0.3327) F_2, 322_ = 2.78 (*p* = 0.0632)	0.912 0.039 0.007 0.017	1.000 0.950 0.244 0.546
	Control; *n* = 200	68.70 ± 10.62	66.68 ± 8.68	67.67 ± 12.74				

Beh—behavioural addictions; *—significant result; M ± SD—mean ± standard deviation; *n*—number of subjects; *p*—statistical significance (ANOVA test); η^2^—effect size (partial eta squared).

**Table 6:** Post hoc comparisons of BIS-AI scores by group and *DAT1* genotype.

*DAT1* and BIS-AI Scale						
	{1} M = 18.95	{2} M = 20.04	{3} M = 25.00	{4} M = 17.95	{5} M = 16.72	{6} M = 17.75
Behavioural addictions 10/10 {1}		0.1204	0.0079 *	0.0782	0.0007 *	0.3177
Behavioural addictions 9/10 {2}			0.0308 *	0.0013 *	0.0001 *	0.0649
Behavioural addictions 9/9 {3}				0.0019 *	0.0003 *	0.0037 *
Control 10/10 {4}					0.0381 *	0.8628
Control 9/10 {5}						0.3960
Control 9/9 {6}						

*—significant statistical differences; M—mean.

## Data Availability

The data presented in this study are available on request from the corresponding author. The data are not publicly available due to privacy concerns.

## Supplementary Materials

The following supporting information can be downloaded at: https://www.mdpi.com/article/10.3390/biomedicines13081852/s1, Figure S1: Gel image showing the results of DAT1 homozygous (10/10, 9/9) and heterozygous (9/10) genotypes.
