# Supplementary figures and images for: Associations Between DAT1 Gene VNTR Polymorphism and Impulsivity Dimensions in Individuals with Behavioural Addictions
Source: Biomedicines. 2025 Jul 30;13(8):1852. doi: 10.3390/biomedicines13081852 (PMC12383520; doi:10.3390/biomedicines13081852)

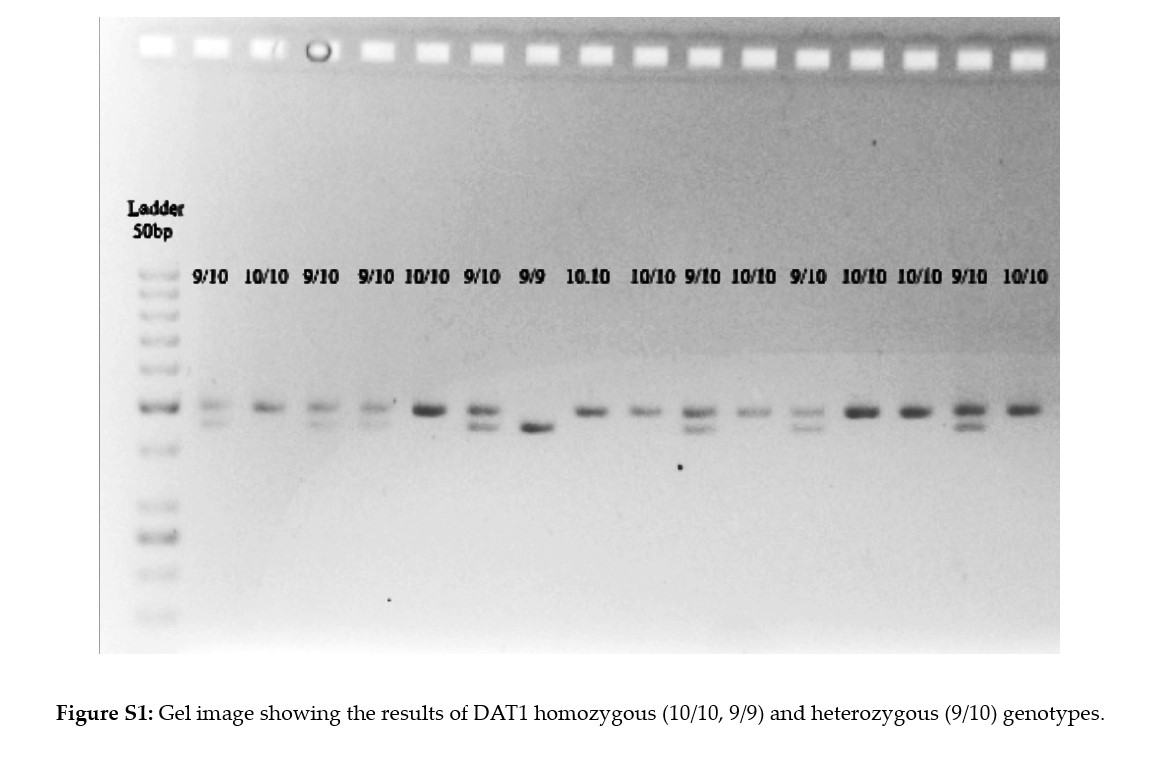

Supplement: Supplementary file 1 [file biomedicines-13-01852-s001.zip › biomedicines-3749745-supplementary.jpg]
